# Rates, Outcomes, and Resource Burden of Extracorporeal Membrane Oxygenation Use in Hospitalizations in the United States During the Pandemic

**DOI:** 10.7759/cureus.54081

**Published:** 2024-02-12

**Authors:** Fidelis Uwumiro, Nuel Otabor, Victory Okpujie, Elsie O Osiogo, Osasumwen F Osemwota, Olawale Abesin, Magaret A Utibe, Nnamdi Ekeh, Arinze E Onyekwe, Oluwatobiloba F Fasoranti-Sowemimo

**Affiliations:** 1 Internal Medicine, Our Lady of Apostles Hospital, Akwanga, NGA; 2 Internal Medicine, University Hospital Coventry and Warwickshire, Coventry, GBR; 3 Internal Medicine, Central Hospital Benin, Benin City, NGA; 4 Internal Medicine, Ahmadu Bello University Teaching Hospital, Zaria, NGA; 5 Internal Medicine, Western Illinois University, Macomb, USA; 6 Internal Medicine, Royal Cornwall Hospital National Health Service (NHS) Trust, Cornwall, GBR; 7 Emergency Medicine, University of Port Harcourt, Choba, NGA; 8 Internal Medicine, Surgicare Hospital, Abuja, NGA; 9 Internal Medicine, Nnamdi Azikiwe University Teaching Hospital, Nnewi, NGA; 10 Medicine and Surgery, Obafemi Awolowo University, Ile-Ife, NGA

**Keywords:** inpatient mortality, hospital resource utilization, nationwide inpatient sample (nis), covid-19, extracorporeal membrane oxygenation support

## Abstract

Introduction: Extracorporeal membrane oxygenation (ECMO) is a lifesaving medical intervention for patients with severe refractory cardiopulmonary dysfunction. This study aims to characterize hospitalizations and resource use burdens associated with ECMO use during the onset of the pandemic.

Methods: We performed a retrospective analysis of ECMO use in United States (US) hospitals between 2019 and 2020, utilizing data from the National Inpatient Sample database. Patient demographics, comorbidities, admission characteristics, inpatient mortality, length of hospital stay (LOS), healthcare costs, and ECMO utilization trends were assessed.

Results: Of the 17,520 hospitalizations analyzed, the most common reasons for admission were diseases and disorders of the circulatory system (40.5%) and diseases and disorders of the respiratory system (31.2%). The average patient age was 52.5 years, with a male predominance (64.2%). Hospitalizations were predominantly for White Americans (59.5%), followed by Blacks (16.3%) and Hispanics (14.8%). Nearly 88.2% of cases were at an extremely high risk of mortality without intervention. Inpatient mortality was significantly associated with Hispanic descent, a higher Charlson Comorbidity Index (CCI) score, age >60 years, and a higher All Patients Refined Diagnosis Related Groups (APRDRG) risk of mortality. Hospitalizations involving ECMO had a significantly higher inpatient mortality rate compared to non-ECMO hospitalizations (43.1% vs. 2.1%, p<0.0001). The mean LOS was 26 days for ECMO hospitalizations, with ECMO initiation occurring approximately five days from admission. ECMO-related hospitalizations often involve over 10 unique procedures, resulting in an average healthcare cost of US$967,647 per hospitalization, totaling US$16.7 billion. Comparatively, non-ECMO hospitalizations had shorter LOS and lower mean costs (mean LOS, 4.7 days, and US$52,659, respectively). ECMO utilization increased significantly from 2019 to 2020, reflecting rising demand for this life-saving therapy.

Conclusion: Compared to non-ECMO hospitalizations, ECMO patients had higher inpatient mortality, associated with Hispanic descent, higher CCI scores, an age >60 years, and a higher APRDRG risk. ECMO hospitalizations had longer stays (26 days) and higher costs (US$967,647 per case, US$16.7 billion total) compared to pre-pandemic levels. ECMO use increased significantly from 2019 to 2020, reflecting rising demand.

## Introduction

The COVID-19 pandemic, with its unprecedented global impact, has exerted immense pressure on healthcare systems worldwide, challenging their capacity to respond to severe respiratory and cardiac complications [1-3]. Among the arsenal of interventions employed to address the clinical complexities associated with this novel virus, extracorporeal membrane oxygenation (ECMO) has emerged as a vital life support modality [4]. While ECMO has long been recognized as an advanced therapy for critical cardiopulmonary failure, its utilization patterns, patient population, and resource implications have experienced a seismic shift in response to the pandemic's unique demands.

ECMO has a rich history that predates the COVID-19 pandemic, with its roots dating back to the early 1970s. Hill et al. reported the first-time use of ECMO for respiratory support in an adult patient with post-traumatic severe respiratory failure. Bartlett et al. went on to successfully use ECMO for neonates experiencing severe respiratory distress in 1975 [5,6]. Initially developed as a modification of cardiopulmonary bypass techniques used in open-heart surgery, ECMO evolved to support patients with severe respiratory or cardiac failure. Over the decades, technological advancements have improved ECMO's efficacy and safety, leading to broader applications in intensive care units. Prior to the COVID-19 pandemic, ECMO was primarily used for severe respiratory failure due to conditions like acute respiratory distress syndrome (ARDS), cardiac arrest, and as a bridge to heart or lung transplantation. There was a noticeable trend toward its increased use, especially in adult patients, driven by improved outcomes and growing expertise in managing complex cases. The 2009 H1N1 influenza pandemic was a pivotal moment, as it demonstrated ECMO's life-saving potential in viral-induced ARDS [7], setting the stage for its extensive use during the COVID-19 pandemic. The pre-pandemic era witnessed a gradual yet steady integration of ECMO into critical care, reflecting its evolving role as an indispensable tool in the management of severe cardiopulmonary conditions.

The pandemic's distinctive challenges, including the sudden influx of critically ill patients and the need for prolonged respiratory support, precipitated a notable increase in ECMO utilization across the United States (US) [8]. This shift raises essential questions about resource allocation, patient outcomes, and indications for ECMO therapy during the pandemic era. While there have been limited studies exploring the changes in ECMO usage, our study specifically aims to evaluate the rates and indications for ECMO utilization and the associated burden on resources during the crucial period of 2019-2020.

The primary goal of our study is to evaluate the rate of ECMO use during the study period. By juxtaposing these findings with pre-existing historical data, we aim to discern the distinct effects of the pandemic on ECMO utilization. Additionally, we endeavor to present an overview of the patient demographics requiring ECMO during this time while assessing the pandemic's influence on patient outcomes following ECMO initiation. A further objective of this study is to examine the burden of resource utilization associated with ECMO. This includes a comparative analysis of non-ECMO hospitalizations and ECMO-related hospitalizations before the onset of the pandemic.

## Materials and methods

Data source

We pooled a dataset comprising 7.3 million inpatient discharge records for the year 2020 from the National Inpatient Sample (NIS) database, a comprehensive database created by the Agency for Healthcare Research and Quality (AHRQ) through the Healthcare Cost and Utilization Project (HCUP). From this pool, we identified adult hospitalizations involving continuous or intraoperative ECMO between January 1st and December 31, 2020. This extensive database records discharges for patients from various payer categories, including Medicare, Medicaid, private insurance, and the uninsured, making it highly representative. The NIS database accurately captures a 20% sample of all hospital admissions across the US, excluding rehabilitation and federal hospitals like the Veterans Affairs hospitals. The NIS sample collects data from 46 states and the District of Columbia, covering approximately 98% of the US population. Each year's dataset typically contains 7-8 million records, each associated with a discharge weight. To ensure representativeness, participating institutions are categorized across five strata based on ownership/control, bed size, teaching status, urban/rural location, and US region. When the weights are properly applied, each year's NIS data provides estimates for approximately 36 million hospital stays. The NIS collects a wide range of data elements. These include details on diagnoses, such as the primary diagnosis and up to 39 secondary diagnoses, the type and timing of procedures, and the total number of procedures performed during each hospitalization. Furthermore, NIS records patient demographics and other resource utilization data such as the length of the hospital stay (LOS), the total number of procedures conducted, and the associated total hospital charges (THC) [9].

Selection criteria and statistical analyses

We employed specific International Classification of Diseases, Tenth Revision, Clinical Modification/Procedure (ICD-10-CM/PCS) codes (5A1522F, 5A15A2F, 5A1522G, 5A1522H, 5A15A2G, and 5A15A2H) to identify adult hospitalizations involving continuous or intraoperative ECMO. We excluded all admissions for patients under the age of 18 and cases not related to COVID-19 to specifically examine ECMO use in pandemic-related hospitalizations. Furthermore, we excluded hospital stays where ECMO was not performed, patients with pre-existing conditions that could confound the analysis and were not directly related to COVID-19 complications, hospital admissions with missing records or data, patients transferred to another facility before ECMO initiation, and readmissions to focus on the initial impact and utilization of ECMO during the pandemic period, ensuring a precise and relevant analysis of the data. Indications for hospitalizations were determined using Major Diagnostic Categories (MDCs). These approximately 25 MDCs each concentrate on a specific organ system, simplifying patient diagnosis classification [10]. Additionally, we identified ECMO indications by extracting ICD-10-CM diagnostic codes (given in the Appendices section) from primary or secondary diagnosis variables within the NIS. Our analysis clusters were based on several critical parameters: geographic region, teaching status, the location of the hospital, bed size, and the ownership of the hospital. To enhance the precision and relevance of our findings, the analysis was further segmented into individual hospitals, each serving as a primary sampling unit. This approach aligns with the guidelines set forth by the HCUP. In addition, each hospital admission in our dataset was associated with a specific discharge weight. The normality of continuous data variables such as age and LOS was assessed using the Kolmogorov-Smirnov test and graphical methods. As recommended by the AHRQ, weighted data were used for all statistical analyses. We accounted for clustering (HOSP_NIS), weighting (DISCWT), and stratification (NIS_STRATUM) within the NIS during this study, ensuring the use of only population-representative data. We utilized survey techniques to assess sociodemographic variables. Specifically, we utilized weighted estimation, complex survey "svy" analysis, and descriptive statistics to analyze and present sociodemographic variables for the hospitalizations included in the study. Continuous variables were presented as means with a standard deviation or median (interquartile range, IQR) for normally and nonnormally distributed data. Categorical variables are presented as absolute numbers with percentages. Missing data was identified using an “mdesc” command within the statistical software. The comorbidity burden was assessed using the Charlson Comorbidity Index (CCI). All statistical analysis was performed using Stata statistical software v.17.0MP (StataCorp LLC, College Station, TX, USA). Trend analysis was performed using the Cochran-Armitage test for trend, with statistical significance set at p<0.05.

Ethical consideration

Since 2012, the AHRQ has enhanced patient and hospital information protection in the NIS dataset by excluding 16 direct identifiers. Furthermore, in adherence to the regulations set forth in the Healthcare Insurance Portability and Accountability Act of 1996, the NIS is designed as a limited dataset, ensuring top-tier data privacy and security standards are upheld [11,12]. Consequently, institutional review board approval was not pursued.

Data availability

We utilized publicly available NIS data for this study. All HCUP databases are available in electronic public repositories and accessible upon email request to the AHRQ at hcup@ahrq.gov.

## Results

Table 1 presents an overview of baseline patient and hospital characteristics, as well as resource utilization for the entire study period (n=17,520). The most frequent reasons for admission were diseases and disorders of the circulatory (cerebrovascular and circulatory) system (40.5%), diseases and disorders of the respiratory system (31.2%), infectious and parasitic diseases (systemic or unspecified sites) (17.8%), injuries, poisonings, and toxic effects of drugs (2.6%), diseases and disorders of the hepatobiliary system and pancreas (1.7%), pregnancy, childbirth, and the puerperium (1.3), diseases and disorders of the blood and blood-forming organs, and immunological disorders (0.3), myeloproliferative diseases and disorders, and poorly differentiated neoplasm (0.2), and multiple significant trauma (0.2).

**Table 1:** Utilization and resource burden of hospitalizations involving the use of ECMO in the US ECMO: extracorporeal membrane oxygenation; LOS: length of hospital stay; US: United States; DRG: diagnosis refined groups; CCI: Charlson Comorbidity Index; THC: total hospital charges; AMI: acute myocardial infarction; CHF: congestive heart failure; COPD: chronic obstructive pulmonary disease; DRG: diagnosis related group; SNF: skilled nursing facility; US$: United States dollar; SD: standard deviation; ARDS: acute respiratory distress syndrome

Variables	ECMO hospitalizations, n (%) unless otherwise specified
Mean age, year ± SD	52.5 ± 0.3
Female	6.272 (35.8)
Male	11,247 (64.2)
Race/ethnicity	
White	10,424 (59.5)
Black	2,856 (16.3)
Hispanic	2,593 (14.8)
Asian or Pacific Islander	666 (3.8)
Native American	193 (1.1)
Other	806 (4.6)
Mean LOS, days ± SD	26 ± 0.7
Mean time to ECMO, days ± SD	4.8 ± 0.2
Mean time from commencement of ECMO to discharge, days ± SD	21.3 ± 0.3
Number of procedures performed	
<2	706 (0.4)
2-4	8,296 (4.7)
5-10	5,712 (32.6)
>10	10,932 (62.4)
Total hospital days	457,704
Mean THC, US$	$967,647 ± $44,406
Aggregate hospital costs, US$	16.7 billion
ECMOs performed by year	
2019	2,646 (15.1)
2020	14,874 (84.9)
Insurance status	
Medicare	4,888 (27.9)
Medicaid	3,767 (21.5)
Private	7,972 (45.5)
Self-pay	876 (5.0)
All patient refined (DRG): risk of mortality	
Minor likelihood of dying	7,008 (0.4)
Moderate likelihood of dying	228 (1.3)
Major likelihood of dying	1,770 (10.1)
Extreme likelihood of dying	15,452 (88.2)
All patient refined (DRG): severity of illness	
Minor loss of function	53 (0.3)
Moderate loss of function	140 (0.8)
Major loss of function	1,174 (6.7)
Extreme loss of function	16,136 (92.1)
CCI	
0	2,943 (16.8)
1	4,433 (25.3)
2	3,749 (21.4)
≥ 3	6,412 (36.6)
Annual median household income (quartile)	
First (0-25th)	5,046 (28.8)
Second (26th-50th)	4,660 (26.6)
Third (51-75th)	4,117 (23.5)
Fourth (76-100th)	3,697 (21.1)
Rural hospital	123 (0.7)
Metropolitan hospital	666 (3.8)
Teaching hospital	16,732 (95.5)
Hospital bed size	
Small	858 (4.9)
Medium	2,278 (13.0)
Large	14,384 (82.1)
Discharge quarter	
First	3,732 (21.3)
Second	3,329 (19.0)
Third	3,889 (22.2)
Fourth	6,588 (37.6)
Hospital region, (%)	
Northeast	3,679 (21.0)
Midwest	4,222 (24.1)
South	6,920 (39.4)
West	2,716 (15.5)
Disposition of the patient (discharge status)	
Routine home discharge	2,032 (11.6)
Transfer to short-term hospital	2,190 (12.5)
Other transfers (SNF, intermediate care facility)	4,047 (23.1)
Home health care	1,612 (9.2)
Against medical advice	70 (0.4)
Died in the hospital	7,551 (43.1)
Weekend admission	3,924 (22.4)
Elective admission	2,172 (12.4)
Primary diagnosis for hospitalization, classified by organ systems	
Diseases and disorders of the circulatory system	7,096 (40.5)
Diseases and disorders of the respiratory system	5,466 (31.2)
Infectious and parasitic diseases (systemic or unspecified sites)	3,119 (17.8)
Injuries, poisonings, and toxic effects of drugs	456 (2.6)
Diseases and disorders of the hepatobiliary system and pancreas	298 (1.7)
Pregnancy, childbirth, and the puerperium	228 (1.3)
Disease and disorders of the blood and blood-forming organs and immunological disorders	53 (0.3)
Myeloproliferative diseases and disorders, and poorly differentiated neoplasm	35 (0.2)
Multiple significant trauma	35 (0.2)
Other diagnoses	736 (4.2)
Specific indications for ECMO	
Acute respiratory failure	12,211 (69.7)
ARDS	41 (23.3)
Cardiac arrest	1,349 (7.7)
Cardiogenic shock	6,552 (37.4)
Covid-19 + respiratory failure	1,699 (9.7)
Pulmonary embolism	193 (1.1)
Severe sepsis	2,522 (14.4)
Comorbidities	
AMI	4,152 (23.7)
CHF	8,427 (48.1)
Peripheral vascular disease	2,085 (11.9)
Renal disease	3,031 (17.3)
Diabetes	2,750 (15.7)
Diabetes + complications	1,927 (11.0)
COPD	3,293 (18.8)
Cerebrovascular disease	2,295 (13.1)
Rheumatoid disease	491 (2.8)
Liver disease (mild)	666 (3.8)
Dementia	53 (0.3)
Cancer	561 (3.2)
Hemiplegia or paraplegia	508 (2.9)
Peptic ulcer disease	456 (2.6)
Metastatic cancer	1,927 (11.0)
Moderate/severe liver disease	438 (2.5)
Metastatic cancer	175 (1.0)
AIDS	70 (0.4)

The average patient age was 52.5 years, with a predominance of males (64.2%). The majority of hospitalizations were for White Americans (59.5%), followed by Blacks (16.3%), Hispanics (14.8%), Asians/Pacific Islanders (3.8%), and Native Americans (1.1%). About 58% of all admissions were for individuals with a CCI score of 2 or higher. Severe loss of function (All Patients Refined Diagnosis Related Groups (APRDRG): severity of illness = 3) was observed in 92.1% of cases, while 88.2% were at an extremely high risk of mortality without intervention. The most prevalent comorbidities included acute MI (23.7%), congestive heart failure (48.1%), peripheral vascular disease (11.9%), renal disease (17.3%), diabetes (26.7%), COPD (18.8%), cerebrovascular disease (13.1%), and metastatic cancer (11.0%) (Table 1).

During the study period, 12,790 patients were discharged alive, while 7,551 (43.1%) died during the index hospitalization. Mortality rates were notably higher in patients with a history of cerebrovascular disease (13.6%; 1,024), congestive heart failure (43.6%; 3,289), and metastatic cancer (12.4%; 938). Conversely, lower mortality rates were observed in hospitalizations involving peripheral vascular disease (1.5%; 112), renal disease (3.2%; 241), diabetes (6.3%; 477), and COPD (5.2%; 393). Most hospitalizations were nonelective weekday admissions and took place at large teaching hospitals, with only 11.6% resulting in routine home discharge. Approximately 12.5% were transferred to short-term hospitals, and 23.1% were transferred to other skilled nursing facilities or intermediate care facilities. Inpatient mortality in the study was significantly associated with Hispanic descent (aOR, 1.35; 95% CI, 1.03-1.77; p=0.031), higher CCI score (aOR, 1.12; 95% CI, 1.07-1.17; p<0.0001), age >60 years (aOR, 1.02; 95% CI, 1.01-1.03; p<0.0001), and higher APRDRG risk of mortality (aOR, 3.61; 95% CI, 2.53-5.15; p<0.0001). The inpatient mortality rate was substantially greater in hospitalizations involving ECMO compared to non-ECMO hospitalizations (43.1% vs. 2.1%; p<0.0001).

The mean LOS was 26 days, with patients receiving ECMO after approximately five days from admission. A significant portion of hospitalizations (62.4%) involved the performance of over 10 unique procedures within the same hospital stay. Collectively, these hospital days added up to 457,704 days and incurred an average healthcare cost burden of US$967,647 ± $44,406 per hospitalization, resulting in a total of US$16.7 billion. Comparatively, other hospitalizations without ECMO lasted fewer days and resulted in lower mean hospital costs (mean LOS, 4.7 days, and US$52,659, respectively).

ECMO use increased significantly over the study period, 2019-2020 (Cochran-Armitage p<0.0001). The number of weighted discharges involving ECMO captured in the NIS database increased from 2,646 in 2019 to 14,874 in 2020.

## Discussion

The findings of this study provide a snapshot of the utilization patterns and resource burden associated with ECMO during the years 2019-2020 in the US. To contextualize these results, it is imperative to compare and contrast them with existing literature, particularly studies conducted prior to 2019. Historically, ECMO (also called extracorporeal life support) has been primarily employed as rescue therapy for patients with severe cardiac and respiratory failure, often as a last resort when conventional treatments fail [13,14]. The Extracorporeal Life Support Organization (ELSO) guidelines stipulate that ECMO should be administered exclusively at tertiary care centers or higher, equipped with neonatal intensive care, pediatric intensive care, and/or adult intensive care units. The center's location should ensure a minimum of six ECMO cases annually and active participation in the ELSO registry. The center's structure should include an ECMO program director, associate directors for specialized ECMO care areas, an ECMO coordinator, and a multidisciplinary team for internal evaluations. Clearly defined policies encompassing indications, contraindications, clinical management, equipment maintenance, therapy termination, and patient follow-up are also mandatory [15,16].

By 2018, a total of 14,205 ECMO runs had been reported in the ELSO registry across over 24,000 centers worldwide. ECMO utilization rates recorded prior to 2019 were significantly lower compared to the remarkable surge observed in US ECMO utilization between 2019 and 2020. Existing literature often portrays ECMO as a niche intervention with a limited scope of application [17]. The marked increase in ECMO use during the pandemic highlights its adaptability as a critical tool in managing a healthcare crisis of this magnitude. The rising trend in the utilization of ECMO during the 2019-2020 pandemic can be attributed to several factors. Primarily, the severe respiratory complications associated with COVID-19, particularly in critically ill patients, necessitate advanced respiratory support, for which ECMO is a viable option. The ECMO's'sbility to oxygenate blood outside the body provided essential life support for patients with ARDS, a common and severe complication of COVID-19. Additionally, the pandemic led to increased awareness and availability of ECMO in hospitals globally as healthcare systems expanded their capacity to manage severe respiratory failure. This expansion included training more staff and acquiring necessary equipment, making ECMO more accessible. Furthermore, evolving clinical guidelines and growing experience among healthcare providers likely contributed to more frequent ECMO deployment as a treatment strategy during this crisis. Recent research has corroborated the increase in ECMO utilization observed in the index study [18,19]. However, there are concerns that centralization of ECMO capacity is lacking in many regions and that the equitable use of ECMO resources remains uneven. In 2020, patients with COVID-19 on ECMO experienced an increase in in-hospital mortality rates, and centers that adopted ECMO early demonstrated a reduced risk compared to those that implemented it later [20].

Considering all possible settings of ECMO utilization, it is probable that its usage during the pandemic displayed a U-shaped pattern [21]. Initially, as case numbers escalated, there was a corresponding increase in ECMO use. However, this trend reversed when healthcare resources became increasingly strained, leading to a decrease in ECMO utilization. As the pandemic's strain on healthcare systems began to diminish, the usage of ECMO might have experienced an uptick. Alternatively, it could have continued to decline if a smaller number of patients met the necessary criteria for ECMO treatment.

Inpatient mortality and resource utilization

The exceptionally high inpatient mortality rate among ECMO patients during the study period is consistent with the severity of conditions necessitating ECMO support and with data reported from previous years of the ELSO registry. Global survival-to-discharge or transfer rates have been reported to be between 45% and 61% for pulmonary and cardiac indications respectively in 2015-2020 [22]. To gauge the pandemic's specific impact, it is vital to compare this mortality rate with pre-pandemic data. ECMO's historical role as a last-resort therapy often associated with poor outcomes in critically ill patients might serve as a benchmark for assessing any changes in patient survival rates over time. In 2020, a systematic review and meta-analysis scrutinizing COVID-19 patients who underwent ECMO revealed a mortality rate of 37% [23]. As the pandemic advanced, treatment approaches also evolved. Amidst these shifts, concurrent studies documented rising mortality rates and extended ECMO durations in COVID-19 patients. The ELSO registry data reflected this change, with ECMO-related COVID-19 mortality climbing from 37% early in 2020 to 52% by year-end [24-26]. In a cohort of 4,227 adult ECMO patients between 2002 and 2012, longer ECMO durations were associated with decreased survival, and more favorable survival rates at 30 days were observed in patients with infection/septic shock, coronary artery bypass graft surgery, and injury as underlying conditions. Extended survival rates reached approximately 20% for infection/septic shock, myocardial infarction/cardiogenic shock, and coronary artery bypass graft surgery patients, while the injury group showed somewhat better survival, exceeding 30% at one year [27].

Resource Use Trends Compared to 1998-2009

The resource burden associated with ECMO hospitalizations, including extended hospital stays and significantly higher healthcare costs, is a consistent finding in this study and may be prohibitive for some patients. A study conducted between 1998 and 2009 analyzed 8,753 admissions involving ECMO utilization. The average LOS for these admissions was 18.3 days. The study found that the average THC per ECMO admission were $344,009. There was a significant increase in cumulative national charges associated with ECMO admissions, rising from $109.0 million in 1998 to $764.7 million in 2009. Additionally, charges per patient and LOS exhibited significant increases. However, the study did not find a statistically significant increase in the number of ECMO admissions over the study period [28]. These findings underscore the considerable financial implications and increasing resource use associated with ECMO therapy during the analyzed period.

In contrast to the earlier study spanning 1998-2009, index findings demonstrate notable changes in ECMO resource utilization. The mean LOS for ECMO-related hospitalizations has increased to 26 days, with ECMO initiation occurring around five days after admission. These hospitalizations often involve a substantial number of procedures, averaging over 10 unique procedures per case. Consequently, the average healthcare cost for ECMO-related hospitalizations has escalated significantly to US$967,647 per admission, resulting in a significantly higher cumulative expenditure of US$16.7 billion even after inflation adjustments. Comparatively, hospitalizations not involving ECMO have exhibited shorter LOS and lower mean costs. Non-ECMO hospitalizations have an average LOS of 4.7 days and an average cost of US$52,659. This stark contrast in resource utilization between ECMO and non-ECMO cases highlights the substantial financial burden associated with ECMO therapy. Furthermore, the index findings reveal a significant increase in ECMO utilization from 2019 to 2020, reflecting a heightened demand for this life-saving intervention. This surge in demand emphasizes the growing recognition of ECMO's critical role in managing severe respiratory and cardiac conditions.

Limitations

As with any study, there are certain limitations that merit acknowledgment. The COVID-19 ICD-10 code (U07.1) was not implemented until April 1, 2020, potentially leading to an underestimation of cases involving ECMO for undiagnosed COVID-19 ARDS. Our approach relied on diagnostic and procedural codes rather than clinical assessments, which introduces the possibility of coding errors. We did not examine the monthly variations in ECMO utilization vis-à-vis the fluctuating COVID-19 surges during the study period. Additionally, due to the nature of NIS data recording discharges rather than individual patients, our study could not ascertain the rates of repeated ECMO interventions. Due to the nature of the study dataset focusing on inpatient ECMO use, the application of ECMO in outpatient settings, such as clinics or home care, and pre-hospital environments, like emergency medical services, was not studied. The findings are therefore not generalizable outside of the inpatient setting. Additionally, post-discharge outcomes, including long-term effects and rehabilitation related to ECMO, fall outside the scope of this inpatient-focused dataset. The index study does not include data from specialized centers that may not be fully represented in the sample, as well as any usage in non-reporting facilities. Lastly, the index study's geographical limitation to the US excludes international ECMO utilization patterns. Recognizing these limitations is essential for a comprehensive understanding of ECMO usage and its broader implications.

## Conclusions

Hospitalizations involving ECMO had a substantially higher inpatient mortality rate than non-ECMO cases. ECMO-related hospitalizations incurred an average hospital cost of US$967,647 per admission, totaling US$16.7 billion. Significantly, ECMO usage experienced a pronounced upsurge from 2019 to 2020, reflecting an increasing demand for this life-saving therapy. The surge in ECMO utilization during the pandemic has profound implications for future healthcare policy and ECMO utilization. This trend underscores the critical role of ECMO in the management of severe respiratory and cardiac failures, particularly in crises. However, it also highlights the need for healthcare systems to adapt to the increased demand for such advanced life-support technologies. Policymakers must consider strategies for expanding ECMO access, ensuring adequate training for healthcare professionals, and investing in infrastructure and technology to support ECMO services. Additionally, the financial implications of rising ECMO use, given its high cost, necessitate the development of cost-effective approaches to ECMO deployment, including criteria for patient selection and protocols for its use, to optimize outcomes and ensure the sustainability of healthcare resources. The lessons learned from the pandemic could drive innovations in policy that balance the immediate benefits of ECMO with long-term healthcare system resilience and equity while avoiding physician availability bias and overutilization of ECMO.

## Appendices

**Table 2:** ICD-10 diagnostic and procedure codes used in the study ICD: international classification of diseases

ICD-10 codes	Code description
5A1522F	Extracorporeal oxygenation, membrane, central
5A15A2F	Extracorporeal oxygenation, membrane, peripheral veno-arterial
5A1522G	Extracorporeal oxygenation, membrane, peripheral veno-venous
5A1522H	Extracorporeal oxygenation, membrane, central, intraoperative
5A15A2G	Extracorporeal oxygenation, membrane, peripheral veno-arterial, intraoperative
5A15A2H	Extracorporeal oxygenation, membrane, peripheral veno-venous, intraoperative
U07.1	COVID-19
A41.9	Sepsis, unspecified organism
I26.01, I126.02, I126.09	Pulmonary embolism
R570	Cardiogenic shock
J96	Respiratory failure
J80	Acute respiratory distress syndrome
I46.9	Cardiac arrest
